# Electro-Therapeutics
*"Practical Electricity in Medicine and Surgery." By G. A. Liebig, jun., Ph.d., and g. H. Rohé, M.D. 380 pp. (F. A. Davis.)


**Published:** 1891-02-07

**Authors:** 


### ELECTRO-THERAPEUTICS.*

In the work before us it has been the aim of the authors to set forth, in a concise manner, the fundamental principles which are involved in the application of electricity to medical and surgical practice. The great advances in electro-therapeutics of the past few years have been unavailable to many of those who have been engaged in general practice during any considerable period, for the reason that most works on this subject presuppose some general knowledge of physics and electricity; for these a handbook of this kind has become a necessity. The text is plain and to the point, and illustrations are freely introduced; thus the manipulation of electrical apparatus, and its practical employment in medicine and surgery, is rendered comparatively easy, while the therapeutical results to be obtained from electricity are fairly stated.

* "Practical Electricity in Medicine and Surgery." By G. A. Liebig, jun., Ph.D., and G. H. Rohé, M.D. 330 pp. (F. A. Davis.)

